# ‘None of us are free until all of us are free’: Introducing collective liberation as a praxis‐oriented framework and concept into social psychology

**DOI:** 10.1111/bjso.70071

**Published:** 2026-03-31

**Authors:** Julia A. Schreiber, Yara Zebian, Mete S. Uysal

**Affiliations:** ^1^ University of Sussex Brighton UK; ^2^ University of Exeter Exeter UK

**Keywords:** allyship, collective action, collective liberation, oppression, resistance, solidarity

## Abstract

Recent years have seen a resurgence of protest and resistance movements worldwide, reminding deep interconnections between struggles for liberation beyond borders, histories and identities. While activists frequently frame these efforts through the lens of collective liberation, this lens remains absent from mainstream social psychology. In this article, we introduce collective liberation as a framework, concept and practice for social psychology. We critically examine the epistemological, methodological and conceptual constraints that have obscured it, and turn to activist expressions and theorizing to articulate three core components central to collective liberation: interlocked systems of oppression, interdependency of individuals and their freedoms, and shared responsibility for their liberation. We situate collective liberation alongside, yet distinct from, existing research constructs in collective action, resistance, allyship and solidarity, blurring the lines between allyship and resistance. Finally, we propose a research agenda that integrates collective liberation into social psychological theory and practice, offering new avenues for studying sustained activism and resistance, cross‐movement solidarity and the psychological processes that support long‐term social change.

"*If you have come here to help me, you are wasting your time, but if you have come because your liberation is bound up with mine, then let us work together.*"

–Lilla Watson (Aboriginal Activist, Academic, & Artist)

The last decade has observed an eruption of protests across cultures, peoples and nations, from riots in Chile and Hong Kong to the George Floyd protests in the United States and the Palestinian Unity Intifada. Although geographically separate and seemingly different in their objectives and grievances, both the tactics of oppression and the popular resistance to oppression have reinforced the interconnectedness of these struggles beyond national borders and agendas (della Porta et al., 1999; della Porta & Tarrow, 2004).

Examples abound. During the police repression of George Floyd's protests, protestors from Palestine and Lebanon shared tips and wrote guides to BLM protestors, detailing their experience with US‐made tear gas and protest safety (@rananazzalh, 2020; @sa0un, 2020). Similarly, many climate activists and queer groups have taken an active stance against the Genocide in Gaza by joining pro‐Palestine solidarity marches (e.g., James, 2024). Past historical struggles have also underscored the interconnected nature of liberatory struggles. For instance, Sarah and Angelina Grimké argued that women could never achieve freedom independently of Black people, and began advocating and organizing for Black liberation (Davis, 1981; Grimké, 1837). Similarly, various demonstrators of the US civil rights movement, such as Bayard Rustin and Martin Luther King, were influenced by the Indian independence movement, often adopting Gandhi's philosophy of nonviolent but disruptive resistance (Afuape & Kerry Oldham, 2022). Comrades from both contexts also actively organized for and participated in one another's liberatory struggles (Afuape & Kerry Oldham, 2022). All these examples illustrate that liberation movements do not happen in silos; they are instead perceived by movement actors to be connected. In doing so, these instances emphasize the necessity of framing and understanding the liberation of oppressed peoples as one, beyond borders and fragmented struggles.

Some recent work in social psychology has started to address the phenomenon of cross‐group alliances in the form of interminority solidarity (e.g., Burson & Godfrey, 2020; Galván‐Hernández et al., 2026; Maxwell et al., 2025; Uysal, Uluğ, et al., 2022) or allyship (e.g., Radke et al., 2020; Uluğ et al., 2024). Yet, we argue that the current angle through which mainstream social psychology looks at cross‐group solidarity is often too simplistic, incomplete and neglects some crucial voices on the ground. For example, while some research theorizes that interminority solidarity comes from perceived similarities of victimization of oppressed groups (e.g., Craig & Richeson, 2012; Vollhardt, 2015), some activist expressions highlight that their motivations go beyond mere perceived similarities. They see their oppressions as interlocked, enabled by the same ideologies and sustained by shared material and tactical resources (e.g., Davis, 1981; Garcia et al., 2025; The Combahee River Collective, 1977). Furthermore, our conceptualization of ingroup and outgroup suggests a single‐axis between the advantaged and disadvantaged, neglecting the complications that intersectional identities bring to such clean binary distinctions. As well, some activists challenge the overemphasis on individual freedoms, noting that, given the interconnectedness of oppressions, it follows that individuals and their liberation are interdependent (e.g., Davis, 1981; Lorde, 1984; The Combahee River Collective, 1977). Finally, in contrast to various psychological models developed through research questions focused on the individual factors that lead to participation in one‐off collective actions (e.g., SIMCA; van Zomeren et al., 2008), activists place more emphasis on the long‐term, inter‐generational contributions of the collective to community and movement building, knowledge and mutual empowerment, emphasizing in the process a shared responsibility for liberation. It follows, then, that their questions are more concerned with the myriad dynamics of community‐building and the psychological and structural dismantling of systems (e.g., Davis, 2016; Martín‐Baró, 1996). In activist spaces, the considerations above are part of a bigger, continuously evolving praxis‐oriented framework that is often referred to as *Collective Liberation*.

In this paper, we introduce *collective liberation* into social psychological research. We first describe what collective liberation is, elaborating on the three components central to a collective liberation framework: (a) *interlocked systems of oppression*, (b) *interdependency of individuals and their freedom* and (c) *shared responsibility for liberation*. Throughout, we situate collective liberation within our field, discussing its relation to similar, yet separate, constructs. We then reflect on its absence from social psychology, challenging the social psychological epistemologies and ideologies that we argue have contributed to its absence. We conclude with a discussion of next steps, including concrete implications for our research practices and a research agenda.

## POSITIONALITY STATEMENT

We do not present the framework of collective liberation and related ideas in this article as something novel we have created, but rather as a constellation of long‐standing insights, practices and commitments developed by movements rooted in Black liberation, Indigenous resistance, queer and feminist struggles, and decolonial thought, particularly those from the Global South and communities of colour in the Global North. Collectively, we (the authors) are trained in the fields of social, political and peace psychology. We are not trained in the fields of community, liberation, or decolonial psychology, and not all of us are from communities that have borne the brunt of colonial and racial violence that necessitated the emergence of these subfields. As such, we recognize the responsibility that comes with engaging in this body of knowledge. In doing so, we aim to bring together strands of this historical, rich praxis that has been ongoing across generations and struggles into social psychological scholarship, where we believe they remain under‐theorized and under‐recognized. Our belief that we could undertake such bridging arises from the convergence of our academic and political backgrounds. Our engagement with the concept of collective liberation emerges primarily from our lived experiences as organizers, activists, collaborators and learners within global and local liberation movements. Our understanding of collective liberation is shaped by sustained dialogue with activists and communities whose practices of resistance have long preceded, and often exceeded, the frameworks of psychological theorizing. It is through these relationships, and our own involvement in collective action, that we began to recognize the conceptual and ethical gaps in how social psychology engages with liberation and resistance. This article is a small attempt to bring this practical and political knowledge into the discipline, while highlighting the long‐overdue recognition owed to the insights produced by activists, organizers and underrepresented researchers within our academic spaces.

## COLLECTIVE LIBERATION: FUNDAMENTAL COMPONENTS

This section aims to provide a theoretical introduction of collective liberation to the social psychological literature through a review of current contributions within academic and activist scholarship. We understand collective liberation as a framework, concept and practice at the same time (see Table 1); thus, we argue that reflecting on collective liberation in our field impacts not only our research questions but also our practices as researchers, both of which we will discuss below. We approach collective liberation as a *framework* that can serve as a broad lens for reinterpreting the social psychology of collective action; as a *concept* that can become a definable unit for systematic examination in future research; and as a *practice* that reflects an embodied set of commitments to be adopted by both activists and researchers. Given the scarcity of scholarship describing and defining collective liberation, as well as the proliferation of this concept within activist circles, we turn to activist literature predominantly in describing what collective liberation is, while also utilizing academic work when deemed relevant. In keeping with the practices we hope our field to uphold, we write this text with the aim of blurring the contrived lines between researchers and activists. We do so by treating equally, and as importantly, the works of activists and academics. In so doing, we aim to challenge the knowledge hierarchy enforced between these two groups, thus also ensuring more novel and grounded insights (for similar approaches, see Sundberg, 2007, as an example).

The activist literature we turned to consisted of blogs, websites, speeches and books by activists and organizers who have spoken about collective liberation. Across our readings of activists’ texts, blogs, websites, speeches and blogs, we identified three concepts often discussed in relation to collective liberation, which we conceptualize as the components, or tenets, that combine to explain what collective liberation is. Based on this, we discuss below the three components that we identified as essential for a grounded understanding of collective liberation across this activist literature: (1) interlocked systems of oppression, (2) interdependence of individuals and their freedom and (3) shared responsibility for liberation. We find that these three components represent the minimal features that must be present for collective liberation.

### Methodology

In order to define collective liberation, we engaged in a conceptual synthesis of some selected texts, speeches and blogs on collective liberation by activists, through which we derived these three most prominently discussed components of collective liberation. Here, we would like to elaborate on how we believe our positionality guided our decision to approach this paper as we have. Not uncommonly, our decision to write this paper came about after endless casual discussions between the authors on their experiences, practices, political education and/or conversations within pro‐Palestinian political organizing circles. As such, we were already enmeshed in discussions around, and some activist literature on, collective liberation (e.g., Davis, 2016). However, so as not to have been solely guided by knowledge from our circles, we explored how other activists discuss collective liberation on the web. We selected the activists’ texts by first searching ‘collective liberation’ on Google and YouTube, which we opted for as we wanted to remain inclusive to the different formats through which activists may decide to express themselves (blog, video, articles). We selected the works that used the term ‘collective liberation’ from the first 2–3 pages, stopping at saturation (*n* = 17). We identified some commonly shared components that built the starting point for this paper, supplementing their description with additional academic and non‐academic readings we had garnered through our knowledge on the subject matter. We then conceptually synthesized the activists’ texts with other activists’ literature that we have come across through our activism, political organizing, and engagement with activists, as well as relevant academic literature. With this approach, we aimed to enrich our theoretical work by integrating both 1) our knowledge and readings and 2) different actors who have engaged with subfacets of these components to varying depth and degree. We recognise that our method is limited insofar as we utilise English‐only texts, and rely on inevitable platform‐specific algorithmic constraints. We acknowledge and treat all of these texts as equally contributing to the ideas that we have discussed in this paper. Lastly, we hope that we have represented the ways that activists and political organizers conceptualize and implement collective liberation within their theory and practice.

### Interlocked Systems of Oppression

We are actively committed to struggling against racial, sexual, heterosexual, and class oppression, and see as our particular task the development of integrated analysis and practice based upon the fact that the major systems of oppression are interlocking. The synthesis of these oppressions creates the conditions of our lives. –The Combahee River Collective, 1977

A core component of collective liberation is recognizing that systems of oppression are interlocked (e.g., Beautiful Trouble, n.d.; DeJesus, 2024). As activist Myisha T. Hill notes (TEDxDelthorneWomen, 2022), we navigate our lives within a ‘matrix of oppression’ where systems such as imperialism, capitalism, colonialism, white supremacy, patriarchy, ableism, the oppression of animals and nature are all intertwined. While other related fields in psychology (Liberation, Decolonial, Black, Community Psychology; e.g., Adams et al., 2015, Prilleltensky, 2003, 2008) have centred oppression, mainstream social psychology predominantly focuses on inequality (David & Derthick, 2017), addressing the *symptoms*, such as unequal power distribution, rather than the systemic origins and beneficiaries of unearned privilege. Even when oppression is studied, our field often overlooks their interconnectedness (e.g., examining sexism without an analysis of racism; for exceptions see Bixby, 2024; Crenshaw, 1989), yet ‘[t]here is no such thing as a single‐issue struggle because we do not live single‐issue lives’ (Lorde, 1984, p. 138). We echo activists who argue that these oppressions cannot, and should not, be treated in isolation, as they sustain and reinforce one another: First, oppressive systems exchange tactics of oppression (e.g., Shahid et al., 2023). Second, they are often rooted in shared histories and ideologies about the world (e.g., Hodson & Dhont, 2023). Third, their interplay disproportionately impacts the already oppressed (i.e., intersectionality; Crenshaw, 1989). Fourth and last, they fragment struggles (e.g., Moss, 2019; Sarkar, 2025). We describe and illustrate these further below, while noting that their expression varies across systems and contexts.

First, these systems of oppression teach and learn concrete tactics of oppression from each other. These tactics are integrated in the political, economic and cultural structures of domination, and can vary from physical force, exploitative laws and practices, and political exclusion, to psychological mechanisms of oppression, such as oppressive narratives (David & Derthick, 2017; Martín‐Baró, 1996; Moane, 2003). While research has addressed the presence of similar tactics and psychological mechanisms across oppressions, it often refrains from questioning why these similarities might exist: they learn from and teach one another. For example, the oppression of Palestinians, Kashmiris and Black people in the United States is highly interlinked (ICNA Council for Social Justice, 2021; Shahid et al., 2023). Specifically, the Israeli Occupation Forces (IOF) and the Israeli police train many US police departments for crowd control and surveillance based on their experiences with oppressing Palestinians, using tactics such as disproportionate physical force (Amnesty International, 2016). Another example is that of India and Israel, who utilize law as an instrument of oppression by enabling nationals to buy occupied land, thus annexing Kashmiri and Palestinian land, respectively (B’Tselem, 2002; Shahid et al., 2023). Hence, mere explanations of interminority solidarity as perceiving similarities and helping an outgroup (e.g., Ball & Branscombe, 2019) might not hold in such cases where oppressed people may (also) perceive their solidarity as resistance against that same oppressor (e.g., Meeusen et al., 2019). For instance, Sundberg (2007) found that activists who learned that the US Army trained the Latin American militaries on torture methods built a collective identity upon a stronger sense of commonality that spanned across struggles, nations and time. Notably, this paper is situated outside of Psychology, in the field of Geography. For this reason, it seems pertinent that social psychological research further explores how, why and when awareness of interlocked systems of oppressions may empower the oppressed, as well as the conditions that distinguish action with the intent to support from action with the intent of co‐resistance.

Second, oppressive structures and practices arise from shared histories and ideologies. For instance, the historical oppression of animals and women (see ecofeminism, e.g., Gaard, 2017), where members of these groups were sacrificed to protect the elite/privileged and community, created the illusion that the acquisition of resources and safety must come with the sacrifice of a ‘subordinate’ group (Kheel, 2008). Similar connections are demonstrated in psychological research that shows that dehumanization stems in the first place from a perception of animals as deserving of fewer rights relative to humans (Hodson & Dhont, 2023). Thus, the claim that humans hold certain cognitions (e.g., zero‐sum thinking), or that they perceive certain groups as subordinate, may be a direct consequence of history, rather than indicative of human nature. Neoliberal beliefs serve as another enabler and illustration of beliefs derived from oppressive systems, such as the oppression of disabled and queer people. Neoliberal and/or capitalistic beliefs reinforce ableism by determining someone's worth based on their productivity and labour (e.g., Russell, 1998). These beliefs also enable the oppression of queer people, insofar as capitalism necessitates reproduction and expansion (e.g., for increasing profits; Copland, 2017). In sum, oppression derives from, and is maintained by, wider global structures of imperialism, colonialism, economic exploitation and norms of competition and homogeneity (Prilleltensky, 2003). In this way, the interconnections of oppressions are not limited to similar tactics, but manifest in the beliefs, perceptions and shared histories that we hold.

While beyond the scope of this paper, we would like to draw readers’ attention to the efforts of some subdisciplines of psychology in acknowledging and describing these oppressive systems as the enablers of structural, inter‐community and interpersonal injustices (e.g., Bronfenbrenner, 1977, 1979, 1986; Foster‐Fishman & Watson, 2017). As one such example, the Bronfenbrenner's Ecological System Theory (1977, 1979, 1986) describes the interrelatedness and connectedness of different systems: they note that microsystems (e.g., family, school, SES), mesosystems (i.e., interactions between microsystems) and exosystems (e.g., government policies, societal and cultural surroundings) are embedded within macrosystems (i.e., overarching ideologies, such as racism) and chronosystems (e.g., developments of systems of oppression over time). In doing so, they emphasize the importance of each of these levels in shaping the immediate surroundings in which humans are embedded. Social psychologists should take a similar approach to other subdisciplines (e.g., community psychology; Riemer et al., 2020) in utilizing such system‐thinking approaches, and exploring the societal, political and historical foundations of cognition, prejudice and oppression (see Muthukrishna et al., 2021, for a similar argument on the importance of history for human cognition).

Third, oppressions also reinforce each other by disproportionately affecting those who hold several oppressed identities (i.e., intersectionality; Crenshaw, 1989), further entrenching the lower status of the oppressed group within the hierarchy of resource distribution (Bixby, 2024). Although many of us are disadvantaged by these systems in some ways—with only a minority benefiting—those with different identities face different material, social and political inequalities. Intersectionality focuses on how the intersection of various oppressions impacts the lived experiences of individuals and communities of oppressed groups; specifically, intersectionality emphasizes that individuals experience oppression to varying degrees based on the multiple social identities they hold (Cole, 2009; The Combahee River Collective, 1977). For instance, a Black woman's experiences of oppression likely include sexism, comparable to other women, but also racism, comparable to other people of colour. That said, Black women will often face discrimination beyond the mere sum of gender and race, given that they hold (at least) two oppressed social identities whose interaction creates unique experiences of oppression (Crenshaw, 1989; The Combahee River Collective, 1977). For example, compared with White women or Black men, Black women are not seen as prototypical representations of their gender or race, which consequentially often renders their voices unnoticed and unheard (Billups et al., 2022; hooks, b., 1981; Hull et al., 1982; Sesko & Biernat, 2010). To this end, oppression cannot be treated as a binary, given various such nuances in the lived experiences of those with different disadvantaged and/or privileged identities.

Lastly, interlocking systems of oppression maintain themselves by fragmenting oppressions and struggles, with the aim of undermining resistance (e.g., Moss, 2019; Oluo, 2025; Women of Color Therapy, 2024). One strategy of fragmentation is the emphasis on scarcity of, and competition over, resources and services, such as education or jobs (Prilleltensky, 2003) or attention in today's algorithmic age (Sarkar, 2025). For example, research showed that oppressed group members, such as Turkish or Moroccan Belgians, who perceive discrimination in the job market and, in turn, a competition over jobs, have more negative attitudes towards other oppressed groups, such as new immigrants (Meeusen et al., 2019). This zero‐sum thinking and competition is, furthermore, enabled by selective political and societal attention: When governments recognize the victimization of one group (e.g., victims of the Holocaust) but not of other groups (e.g., victims of [settler] colonialism), the neglected victim group will further emphasize their suffering as more severe (i.e., competitive victimhood; De Guissmé & Licata, 2017; Noor et al., 2012). As a result, many people might not see the connections between oppressions, might not engage in collective action for other causes, and may even perceive other causes as competition (Galván‐Hernández et al., 2026; Maxwell et al., 2025; Vollhardt, 2015). On the ground, this may engender fewer effective movements given smaller numbers, less visibility and resource investments in competition rather than joint resistance against a common oppressor.

### Interdependence of individuals and their freedom

We are caught in an inescapable network of mutuality, tied in a single garment of destiny. Whatever affects one directly, affects all indirectly–Martin Luther King Jr. (1963)

Collective liberation moves away from (hyper‐)individualism and universal absolutism, instead focusing on the interdependence of individuals and their freedom (e.g., enfleshed, n.d.; Oluo, 2025; Pai, 2022; Salih, 2024). Our relationships are reciprocal, and the survival of peoples would not be possible without the co‐resistance of other peoples. Since systems of oppression are interlocked and learn from each other, ‘other’ people's oppression (in)directly affects all our freedom; consequently, the liberation of ‘the other’ is not possible without the liberation of all oppressed peoples. A very timely example of the popular consciousness to this sentiment is the rise of the chant *‘none of us are free until Palestine is free’* within pro‐Palestinian solidarity circles—a rendition of Emma Lazarus's (1883) original slogan, *‘until we are all free, we are none of us free’*.

Various scholars and activists have differentially discussed the interconnectedness of our freedoms. First, since systems of oppressions are interconnected, dismantling part of one oppressive system destabilizes and weakens other oppressive systems, as it proves more difficult to uphold a system that has already been exposed as unjust for others. It inevitably follows that our freedoms, which are restricted by these same forces of oppression, would be similarly interconnected. Second, some highlight that the interconnectedness of one's lived experience of oppressions (i.e., intersectionality) renders it impossible to wholly liberate one group without having liberated other groups. Women, as one such example, are only truly liberated when *every* woman is liberated (e.g., Lorde, 1984), necessitating that other oppressions, such as racism and classism, be redressed for a Black woman or a woman of low socio‐economic background to be included in this liberation.

It follows that such a mindset of shared fate and interconnected liberation might explain the tendency of various oppressed groups to reject a competition of resources and sufferings (Vollhardt et al., 2021, 2023), instead acting in solidarity with one another and centring the experiences of people with several intersecting oppressed identities. For them, joining one struggle means not only working towards the liberation of one certain group but working towards the liberation of all other groups simultaneously. Solidarity also becomes instrumental in reaching wider audiences and mobilizing greater numbers, thus gaining closer societal and political attention for movements and maintaining their relevance over time (Garcia et al., 2025; Zia, 2020).

What's more, the liberation of all is a collective feat rather than an effort dependent on the courage of exceptional individuals, and spans across history and struggles. As Angela Davis (2016) states:

Progressive struggles—whether they are focused on racism, repression, poverty, or other issues—are doomed to fail if they do not also attempt to develop a consciousness of the insidious promotion of capitalist individualism. Even as Nelson Mandela always insisted that his accomplishments were collective, always also achieved by the men and women who were his comrades, the media attempted to sanctify him as a heroic individual. (p. 1)

This understanding highlights that every single one of us can become an actor of change (e.g., Pearson, n.d.), and that our determination to be active might be shaped by the collective, rather than by our individual predispositions (for a similar criticism see Reddy & Amer, 2023). In other words, our movements are dependent on the contributions to, and exchange of knowledge across individuals, which in turn empowers the development of innovative, flexible approaches that challenge oppression in all its current and evolving forms (e.g., Garcia et al., 2025). More so, collective liberation acknowledges struggles for social justice as inter‐generational, often at the expense of present generations whose actors may not necessarily benefit during their own lifetime (e.g., APALA, n.d.). Consequently, collective liberation emphasizes the dynamic nature of liberation as a constant learning process that goes beyond personal gains, transcending our mainstream social psychological understanding of political efficacy (Agostini & van Zomeren, 2021).

### Shared responsibility for liberation

[Collective liberation] means acknowledging that when it comes to issues of equity and justice, we all have learning to do. It means owning the contributions we make to systems of oppression and how we benefit from those systems. It means recognizing that in order to create spaces that honor the humanity and brilliance of everyone, we must start with ourselves. It means investing in our own and one another's transformation. –Staley and Leonardi (2022)

Understanding our liberation as shared, collective and rooted in community‐building by default centres our responsibilities as individuals within said community (e.g., Hernandez, 2021; Jordan, 2024). It demands that we confront the way we maintain and contribute to the systems we seek to dismantle. As most of us are oppressed—albeit to varying degrees—by interlocking systems of oppression, each of us is, to some extent, complicit in maintaining these same systems. Collective liberation requires placing as much value in breaking social, political oppression as well as personal, psychological oppression (Martín‐Baró, 1996). Whereas political oppression refers to the exertion of "material, legal, military, economic, and/or other social barriers to the fulfilment of self‐determination, distributive justice, and democratic participation" (Prilleltensky, 2008; p. 127), psychological oppression can be defined as an individual's internalized beliefs of their group's inferiority and helplessness, and consequently, their perception that less access to resources and political and societal participation is deserved (Prilleltensky, 2008). This reflection on oppressive systems, and the responsibility and action of dismantling them, have been referred to by previous literature as critical consciousness (Fanon, 1961; Freire, 1970; Moane, 2003; Prilleltensky, 2003; Watts et al., 2011).

At this point, we find it important to emphasize that our focus on highlighting the interconnectedness of struggles and oppression of all peoples, and therefore this shared responsibility, is not to erase, minimize, or flatten the experiences of oppression across peoples. Similarly, we refuse a framing that deems all peoples as equally implicated (or equally oppressed). Rather, we emphasize the distinct forms of responsibility placed onto those with greater systematic privilege, given their direct benefit from that oppression (The Combahee River Collective, 1977). We aim to add necessary complexity to our understanding of social change, but not to equate the disadvantage experienced by intersectional individuals belonging to historically privileged groups (e.g., white, cis‐gender, men) with the oppression of historically under‐privileged and/or previously colonized groups. Acknowledging multi‐dimensional oppression does not deny systematic privilege but rather calls for a more nuanced analysis of the intersection of powers and therefore resistance. This is why we frame collective liberation as not just a potential concept (for empirical research) but also a potential framework, or lens, through which to engage with our research. And this is why we acknowledge that a responsibility for liberation, and for resisting one's own complicity, will look differently among people and groups, often shaped by their positionality, historical identity and access to resources.

Yet at its core, such a responsibility will necessitate an openness to, and proactive partaking in, continuous self‐criticism, reflection and unlearning of the oppressive structures and subsequent thinking and behaviours they internalize within us (e.g., DeJesus, 2024). Such is done by considering our privilege(s) (through the benefits we gain from these systems), the institutions and practices we support, the prejudices we hold, the social norms we subscribe to, the beliefs we take for granted, and the actions we (do not) partake in to liberate ourselves and others. This work is often a messy process of making mistakes, learning, reflection and humility; it is not a linear job. It requires mutual accountability, where feedback, critique and repair are not only possible but expected—a space void of cancel culture (Brown, 2021) and abundant with cultures of care (Mingus, 2011). Our practices, emotions and identities are shaped by dominant ideologies, both on the side of the oppressor (e.g., prejudice, coloniality) and the oppressed (e.g., internalized oppression, learned helplessness; e.g., Fanon, 1961; Rivera Pichardo et al., 2022, 2023). If we are not conscious of this shaping, we risk reproducing harm even in spaces dedicated to justice. Activist Angela Davis (2016) reiterated this, dutifully noting that "what we often assume belongs most intimately to ourselves and to our emotional life has been produced elsewhere and has been recruited to do the work of racism and repression" (p. 151). Our inability or refusal to reflect on our own complicity effectively impels us into maintaining the very structures we oppose, whether intentionally or unintentionally. This framing turns liberation into both an inward and outward practice: resisting oppressive structures in society while also dismantling their internalized forms within our thinking, relationships and everyday practices.

### Boundary conditions of collective liberation

Cognizant that our audience is primarily researchers, we find it also important to outline our perceived boundary conditions for collective liberation. We emphasize these as provisional boundary conditions, based on our conceptual synthesis of selected activist texts as well as other readings we have selected in tandem with our beliefs and interests. We see these boundaries as an attempt to provide more conceptual clarity to enhance the construct's falsifiability and researchability. First, given the prevalence of the three components in activists’ understanding of collective liberation, we note that all three components must be observed by the researcher across conversations with interlocutors for a phenomenon to be understood as collective liberation. An awareness of the interlocked systems of oppression and power (Component 1) would need to co‐exist with a realization that our resistance movements against these systems are interdependent (Component 2) and a recognition that every individual holds a responsibility for our collective liberation (Component 3). Second, a phenomenon would not qualify as collective liberation if it is used to erroneously flatten or equalize oppressions across different groups and/or group members. As mentioned above, understanding the interconnectedness of oppressions and liberatory struggles does not minimize the greater systematic advantage given to historically privileged groups. Third, for a phenomenon to qualify as collective liberation, a systems‐level approach to understanding injustice/oppression must be observed by the researcher across conversations with interlocutors. Awareness of the intersection of individualized experiences and identities, as it is often erroneously prioritized by liberal identity politics over system‐level thinking, is not sufficient for collective liberation without a recognition and analysis of the intersection of oppressive structures and vulnerabilities created by these interconnected systems.

**TABLE 1:** Collective liberation as framework, concept and practice.

What does collective liberation contribute as a framework, a concept and a practice?	
Framework	Collective liberation provides a broad theoretical lens through which to understand social change, oppression, resistance, and solidarity. Rather than focusing on isolated actors, single actions or discrete events, it foregrounds the systemic and interlocked nature of oppression, the interdependence of people's freedoms, and the shared responsibility for dismantling oppressive structures. This framework shifts analytical attention from improving relative conditions within existing systems to examining long‐term, collective processes aimed at transforming those systems themselves, it also prompts us to reshape our understanding of solidarity and resistance, and blur the contrived lines in between. In doing so, collective liberation reshapes what counts as meaningful resistance, how movements are related to one another across time and context and what the ultimate aims of social change research should be. As researchers, this compels us to reflect and understand the (historical) connections of oppressions to contribute meaningfully to structural and psychological liberation, and account for the nuances in people's experiences and understandings of their multi‐axis identities. Among political and community organizers, collective liberation is utilized as a framework for political analysis and a lens through which to understand the political world; it is also used as a guide towards engaging with struggles, defining objectives and deciding on tactics and strategies.
Concept	As a concept, collective liberation offers a new construct and, with it, a multitude of research directions. First, collective liberation needs more empirical attention itself, in order to explore how activists understand collective liberation, what sets of beliefs and perceptions can be associated with collective liberation, and how they align their practices to this mindset. Furthermore, research could look into how people build a collective liberation mindset, and what might hinder this development of beliefs. For outcomes, research could look into whether collective liberation might open new opportunities for alliances and movements (e.g., making them more sustainable and attracting wider masses), might be able to empower the oppressed, or might come with some drawbacks (e.g., tensions within movements).
Practice	For researchers, practicing collective liberation necessitates that we reflect on how our practices can serve the most oppressed in pursuit of liberation. This includes acknowledging that researchers (i.e., humans) are incapable of neutrality, and subsequently, that the myth of neutrality only benefits the oppressor. Our writings, institutions and associations should reflect the values that our field should uphold. It further means that we should cognizantly centre the oppressed, across our methodologies, practices and theorizing, in pursuit of more grounded, justice‐oriented, liberatory research. This can happen by centring the knowledge and experiences of oppressed activists and researchers: On the one hand, by treating them as leaders or collaborators of projects, rather than as data collectors for understudied countries and populations. On the other hand, by critically reflecting on our research methodologies/epistemologies, conducting more participatory research and valuing different forms of knowledge, and methods of knowledge production, equally. Political and community organizers practice collective liberation in the strategies through which they fight for their struggles and the ways that they show up for their struggles. More so, they practice collective liberation through the radical acts of care and love they embed within their circles and the communities they build. In doing so, practicing collective liberation becomes a form of constructive resistance for organizers.

## WHY IS COLLECTIVE LIBERATION MISSING FROM SOCIAL PSYCHOLOGY?

Collective liberation is glaringly missing from social psychological research and discourse. We argue that social psychology has not created the space for collective liberation to be discerned by constraining the epistemological, methodological and conceptual tools that enable such systems‐level thinking. We discuss these constraints below while mindful of their interdependency. We focus our discussion on the social psychology of collective action, resistance, allyship and solidarity.

Social psychological research on social change has limited itself to (Vollhardt et al., 2020), and extensively studied (Agostini & van Zomeren, 2021; van Zomeren et al., 2008), one form of collective and overt resistance, namely *collective action*. In so doing, scholars have recurrently adopted Wright et al.'s (1990, p. 995) most cited definition of collective action as action that is undertaken by an individual anytime they are "acting as a representative of the group and the action is directed at improving the condition of the entire group". Yet we argue that this definition may imply that the purpose of social change is not one of liberating oppressed groups from systems of oppression, but one of improving the conditions of those oppressed groups, relative to each other, within the current hierarchical system, thereby often limiting the field's conceptualization of social change.

In fact, one of the historical foundations of collective action literature perfectly illustrates this understanding of social change: Relative Deprivation Theory (RDT; Runciman, 1966; van Zomeren et al., 2008). A study of US military camps found that African American soldiers in Southern camps adjusted better to army life than those in Northern camps, where discrimination was less severe (Stouffer et al., 1949). It seemed that African American soldiers in the South perceived greater advantages over African American civilians in the South than Northern soldiers did over civilians in the North. Stouffer and colleagues coined the term *relative deprivation* to explain this pattern (Runciman, 1966), which became central to theorizing predictors of collective action intentions (for review, see van Zomeren et al., 2008). Stouffer and colleagues’ case study, as well as majority of subsequent research on collective action that builds on RDT, has led the field to conceptualize collective action as a battle of oppressions (of ‘who has it worse’) among nonetheless oppressed populations, leading social psychologists to ask research questions that do not fully capture the complexity of the situation. For example, while African Americans in the Southern camps may have perceived their treatment as less unjust than those in the North, it remains the case that soldiers in both camps were treated unjustly, nevertheless. The focus on the *relative conditions* of groups, rather than on the political and psychological oppressions that contribute to accepting an oppressive reality, leaves the systems of oppression that enact this discrimination intact. After finding out that Southern camp soldiers perceived less discrimination, research might have instead explored how Southern soldiers’ perceptions (that they are treated less unjustly, despite evidence to the contrary) were shaped by structural, systematic and contextual conditions. This could have also been compared with the conditions of the Northern soldiers. It would also have been possible to explore how critical consciousness can be built among Southern soldiers, to emphasize their oppression and encourage their resistance.

We focus on RDT given its foundational contribution to the development of collective action research, yet it is one of many such examples that underscore a main reason for the absence of collective liberation: the research questions social psychologists tend to ask and the ideologies that give rise to them. Formulating research questions grounded in activist experience and beneficial for activists will often require participatory action research approaches (Cornish et al., 2023) by researchers willing to forgo their own research agendas and be driven by the needs and curiosities of community organizers first. However, such approaches are hitherto less utilized in social psychology. This is likely due to its over‐reliance on quantitative research in pursuit of scientific credibility and the myth of objective knowledge (Parker, 1989), thereby contributing to the field's oft‐criticized limited repertoire of methodologies (Adra et al., 2025). Concerning qualitative research, we distinguish between qualitative research *on* activists and qualitative research *for* and *with* activists. While social psychologists have been increasingly turning to qualitative research in the form of interviews and focus groups, more participatory practices remain absent. Such practices become possible through building strong, symbiotic relationships with activists (not just as researcher‐activist, but as oppressed peoples working towards one another's liberation), and through researchers’ lived experience as activists and as active members of political/community organizing circles. This is exemplified through collective liberation, which is prevalently discussed among organizing circles (where the authors of this paper have come across these conversations), but is rarely a point of conversation across social psychological literature on social change.

Given these constraints, we argue that our research has often drifted away from its purpose of contributing to people's liberatory emancipation from oppression and has, at times, come to function as an ideological apparatus that reinforces the status quo. While framed as progressive, we see these as selective improvements that legitimize the system by appearing to address injustice yet keeping the structure of oppression entirely intact (see also Prilleltensky & Gonick, 1996, for a similar argument). The result is research that makes life for a traditionally disadvantaged group under oppression more bearable while maintaining their oppression, at best. At worst, it makes life more unbearable for the oppressed and more advantageous for the oppressors, reinforcing this relative hierarchy.

Furthermore, most existing research has treated resistance as a static event rather than a dynamic and continuing process (for exceptions see Drury & Reicher, 2009; Obradović & Ivanović, 2026; Vestergren et al., 2018; Vestergren & Acar, 2023). One way this is achieved is through the consistent exploration of collective action as an end goal by its own right, rather than one among many tactics in a repertoire of resistance strategies (Marazzi & Vollhardt, 2024; Tilly, 1993; Vollhardt et al., 2020). Another way is through mainstream social psychological research's predominant focus on the antecedents for engaging in a collective action, namely research that is exploring, replicating, or extending Social Identity Model of Collective Action (SIMCA; van Zomeren et al., 2008). The abundant social psychological research utilizing SIMCA emphasizes the importance of political identification, efficacy beliefs and perceived/affective injustice for predicting (intentions to) engage in collective actions, such as protests or signing a petition (e.g., Chan, 2017; Judge et al., 2022; van Zomeren et al., 2008). Subsequent research suggests separate pathways for different types of political participation (e.g., anger leading to normative action and contempt leading to non‐normative action; Tausch et al., 2011); in doing so, it categorizes normative and non‐normative political engagement as static and unchangeable, as noted by previous scholars (e.g., Uysal et al., 2024; Zhu & Chou, 2022). This is unlike dynamic models, such as the Elaborated Social Identity Model (ESIM; Drury & Reicher, 2005, 2009), which suggest that dimensions of one's identity, along with perceived social norms (e.g., surrounding violence), can change in response to the evolving relationship between oppressor and oppressed.

Indeed, most static models may inadvertently contribute to individualized decision‐making models, leaving little room for theorizing liberation as an interdependent, evolving process of dismantling systems of oppression. This is not to say that studying the conditions that lead to a one‐off act of resistance is in vain; on the contrary, this is particularly necessary given the nascency of a psychology of resistance and our lack of understanding of acts of resistance that are not collective actions (Bou Zeineddine & Vollhardt, 2024). Yet our ensuing theorizing suffers without a conceptualization of social change as a process, and of one‐off acts as situated within a dynamic and ongoing temporal movement spanning across oppressed peoples and causes. It precludes scholars’ ability to consider the interactions between groups and between movements, as well as the long‐term psychological processes required for social change, such as the ongoing role of critical consciousness in engaging in and sustaining collective action (see Watts & Hipolito‐Delgado, 2015).

With all of that said, we find it equally necessary to acknowledge the research that we believe to be challenging this mainstream, whether conceptually or methodologically. For instance, some research looked into the reasons behind movement diffusion (Drury et al., 2025). Others investigated the psychological changes experienced through participation in collective action, recognizing the importance of sustained and consistent participation (Burrows et al., 2023; Drury & Reicher, 1999; Vestergren et al., 2018, 2019). Yet this research remains limited in scope and often stops short of situating these psychological changes within broader, long‐term struggles for liberation across time and space.

## TOWARDS A SOCIAL PSYCHOLOGY OF COLLECTIVE LIBERATION

We have described the three components of collective liberation and outlined likely reasons for its absence from the social psychological literature. Throughout, we noted points of convergence and divergence with existing research. Here, we move beyond critique towards a research agenda that asks: What might it look like to build a social psychology of collective liberation?

Having recognized the limitations of mainstream quantitative approaches, future research on social change must adopt under‐utilized qualitative and quantitative practices that allow for complexity. Quantitative work should embrace approaches that account for heterogeneity (e.g., person‐centred approaches; Thomas et al., 2024) or dynamic processes (e.g., agent‐based models or multi‐level system models; Bou Zeineddine & Leach, 2021). While qualitative research is growing, it remains dominated by interviews and focus groups. These are indeed core qualitative tools, yet researchers should also complement them with participatory and ethnographic methods such as observation or photovoice (Dixon & Durrheim, 2003) applied within a grounded, participatory and horizontal manner. Furthermore, researchers can make use of already existing detailed documentations (e.g., historical documents, narrative biographies) to understand the dynamics of movements and the foundations and developments of our oppressive cognitions (see Drury et al., 2025; Muthukrishna et al., 2021). Moreover, such research does not lend itself to silos, and necessitates a drive for and proactivity towards reading beyond our discipline and practising interdisciplinary research within our readings and writing. With this in mind, we suggest future research directions for a social psychology of collective liberation.

First, we reiterate our turn to activists’ texts, speeches, websites and books in describing the components of collective liberation. The purpose of this undertaking was a humble attempt at merely introducing collective liberation into social psychology. That said, grounded and inclusive research on collective liberation must back up and explore how activists make sense of the concept of collective liberation. This is not to say that the components we developed are erroneous, but that a more in‐depth empirical engagement of primary data with activists, who adopt a collective liberation framework within their practices and theorizing, is necessary to understand and explore the tenets of collective liberation through first‐hand, in‐depth accounts. Similarly, it would be important to explore how these activists practice and incorporate the tenets of collective liberation within their day‐to‐day organizing practices. This could include more research on how knowledge within and across movements is generated over time, and how this shapes the individuals’ beliefs and perceptions. For instance, what forms of knowledge do newer activists gain from more experienced activists, or previous generations of activists (via archival data)? How does this knowledge shape these activists’ perceptions and approaches towards the struggle?

In doing so, we reiterate the importance of conceptualizing social movements as a process rather than an event/act. Such a conceptualization of movements as a collective and long‐term process may explain gaps in our theorizing on why people may act despite low political efficacy (Acar et al., 2024). Currently, efficacy is often measured as efficacy to reach group goals that focus on specific changes/events (e.g., stopping higher education financial cuts; van Zomeren et al., 2013). Other work has already expanded this concept of efficacy and pointed out that people are also motivated to join collective action when they believe it is effective in strengthening the identity of the protesting group by demonstrating the group's values and building an oppositional movement (i.e., identity consolidation efficacy; Ayanian et al., 2021; Saab et al., 2015). Yet, people might actually perceive their actions as effective in engendering political change despite a lack of belief in the efficacy of their own actions for immediate outcomes (e.g., Bleh et al., 2025). When change is perceived as slow, people can still believe that their actions contribute to the wider movement, even if the outcome will not be achieved in one's own lifetime. Moreover, examining the role of collective liberation as a framework and mindset guiding people's prefigurative practices may be a valuable endeavour in social psychology's emerging sub‐field of prefiguration (Clarke & Drury, 2025; Vestergren et al., 2025), in light of some communities that practice collective liberation by centring power within the community and creating systems of care outside of the state (eragoda, 2024).

Concerning allyship and solidarity, we first reflect on some challenges we had writing and thinking through the integration of this framework into social psychology: We struggled with mainstream social psychology's frequent categorization of groups into disadvantaged vs. advantaged and other similar iterations (e.g., minority vs. majority), both at the level of the specific binary, and its underlying assumptions (see Dixon et al., 2020), but also at the level of terminology. Questions arose: Why have we chosen to dichotomize the different actors surrounding an unjust scenario? Why have we chosen to assume that there is a disadvantaged and an advantaged, and how have we decided who is whom? Why have we chosen the terminology of advantage as opposed to that of oppression?

In thinking through the idea of collective liberation, we are prompted—as researchers—to move away from seeing interminority solidarity or allyship merely as helping an outgroup to improve their conditions. While previous work has emphasized that these actions can be based on different motives (Louis et al., 2019; Radke et al., 2020), they currently do not account for members of these groups who might perceive their actions as *co‐resisting* (i.e., resisting against the same oppressive systems) to reach their shared liberation (Schreiber et al., 2025).

Such a framework thus blurs the academic lines that scholars have proposed between allyship, (interminority) solidarity, collective action and resistance. By understanding the different forms of injustice across the world as manifestations of the same system of oppression, and subsequently the liberation of oppressed peoples as one, it stands to question whether—as an example—political organizers for justice to Grenfell^1^ survivors/victims are only acting ‘in support’ when joining and co‐organizing actions against the UK government's complicity in the ongoing genocide in Gaza. We invite readers to think with us, and with activists, about the following musings: When Grenfell tower activists organize against their council for its complicity in Israel's genocide, are they merely in solidarity with Palestinians, or are they resisting themselves, from their own land, against a system that has simultaneously killed their own in a fire and bolstered an imperialist project in the SWANA region? In treating the Grenfell fire as separate from the genocide of Palestinians within our conceptualization and investigations, are we, as scholars, inadvertently perpetuating the oppressors’ efforts to fragment our struggles and thereby weaken us through isolation?

We use Grenfell × Palestine to ground our suggestions, noting that these questions would apply similarly to other struggles. The fragmentation of struggles, and oppression more generally, is seldom just material and psychological, it is also intellectual. As researchers, we can either uphold that oppression through our scholarship or service our knowledge towards social change—in this case by building critical consciousness of the interconnected nature of our oppressions. We call for allyship and solidarity researchers to struggle with these conceptual dilemmas and what they mean for their conceptualizations and research practices. For instance, questioning the possibility that people perceive their liberations to be interdependent acknowledges that those in solidarity may not perceive themselves to be merely fighting for the rights of a different group. By questioning the underlying motives behind participation, researchers may differentiate the circumstances under which one might perceive themselves to be supporting vs. co‐resisting with a group. For this, we might, for instance, examine why and when collectives for one struggle organize to engage collectively in ‘another's’ struggle (e.g., via having the collective represented in a march). For instance, Prilleltensky (2003, 2008) provides a blueprint of questions to consider for achieving epistemic validity (i.e., reflecting and integrating knowledge of psychopolitical dynamics of oppression in our research) and transformative validity (i.e., how does our knowledge production/interventions promote liberation) in our research. Moreover, people's engagement in one struggle might help to inform their resistance in other struggles, and in turn, reinforce liberation processes on several fronts simultaneously. For example, solidarity for Palestine in Arab countries spread discussion about resisting authoritarian structures in their own contexts (Kurd, 2022). In this way, researchers are urged to examine how actively engaging in one struggle might inform our engagement in seemingly separate struggles.

### Research agenda by components

Although we suggest that these components combine to explain the framework of collective liberation rather than acting as isolated constructs, we acknowledge that researching collective liberation benefits from understanding each of these components, which are themselves under‐researched in social psychology. In what follows, we discuss future research applicable to each component described above, while mindful that these concepts and subsequent research on them are not, and must not be, treated as mutually exclusive.

Recognizing that systems of oppression are interlocked opens a wide array of research questions that social psychology is yet to tap into. The political actors that enact and sustain these seemingly separate oppressions share a belief system and common worldviews, regardless of their explicit communication with one another. This adds another element to understanding political elites, and power, that is worthy of investigation. Understanding the neoliberal, far‐right and/or imperialist ideologies that give rise to such systems of oppression is an important undertaking for the field. Little research has been heading in this direction (e.g., Bettache, 2024). Ultimately, the objective of this research direction should be to understand these ideologies to the extent necessary for dismantling them and the ensuing systems they engender. Causal mechanisms in which the interlocked nature of oppressive systems, and awareness of that interlocking, shape specific forms of resistance, efficacy beliefs, belonging, or diffusion across movements, times and places remain underspecified due to limited research. The insights we offer here are grounded in a preliminary analysis of activists’ discourses, and future work should extend this effort by examining activists’ accounts and lived experiences in greater depth, as well as empirically testing the causal pathways through which awareness of interlocking systems shapes these outcomes. For future research into these mechanisms, we can draw inspiration from Community Psychology in how to approach research that integrates power dynamics into social and political psychological theorizing (Prilleltensky, 2003, 2008).

At the level of the oppressed and their own liberation, social psychology must follow suit from its more critical counterparts (e.g., liberation psychology) in exploring how to develop a critical consciousness (Burson & Godfrey, 2020; Freire, 1970; Watts et al., 2011) and an appraisal of these systems as interlocked among those oppressed. Researchers may decide to investigate how critical consciousness might encourage participation in, or organisation of, different forms of resistance across struggles (Diemer et al., 2021), and whether and how it can protect the psyche from certain acts of repression (e.g., reducing competitive victimhood). Beyond mere research, such an appraisal will prove useful in refuting common arguments made by administrations of the academic institutions employing us and the psychological associations claiming to represent us (e.g., European Association of Social Psychology, International Society of Political Psychology). As various researchers and lecturers rushed to organize in solidarity with pro‐Palestinian encampments and against unprecedented levels of academic repression, many were met with whataboutery, defined as a rhetorical tactic aimed to redirect attention from the issue at hand to another issue, in an attempt to weaken advocacy for the former issue (Bowell, 2023). Commonly, top‐down criticisms against expressing solidarity with Palestinians, and Palestinian scholars, would claim: *What about every other oppressed group in the world?* Adopting a framework of collective liberation is thus necessary not only for our research but also for our own political organizing and worldview, within and beyond academia.

Overall, collective liberation highlights the necessity of constant unlearning and the awareness that liberation happens on different levels, and thus should go beyond the analysis of overt strategies of resistance (i.e., collective action), accounting for the before and aftercare of the frontlines (eragoda, 2024). Consequently, collective action and resistance research should expand their focus on different overt and covert forms of action on the individual and collective level before, during and after instances of mass mobilization. Moreover, acknowledging that we are all to some extent complicit centres the shared responsibility that we hold in actively liberating ourselves from these systems. Thus, collective liberation should increase our feelings of moral obligation–a concept with high predictive power for collective action (Ayanian et al., 2021; Uysal, Acar, et al., 2022). Research may explore how fostering a mindset of collective liberation might increase collective action, through a heightening moral obligation that is struggle‐unspecific.

### Potential challenges in practising collective liberation

Although we predominantly focus in our paper on the positive contributions of collective liberation, we find it important to note that a collective liberation framework may often be a source of tension when practised within organizing circles. Organizing with other groups fighting for a similar struggle often already comes with its own challenges, which are only compounded when struggles multiply. This, accompanied by the inevitable risk of activist burnout (Gauditz, 2025), necessitates that activist groups have conversations about which struggles to prioritize their time, capacity and skills for, and which may, as a result, need to be sidelined. The extent to which activists decide to (dis‐)engage with other struggles, and the choice of which struggles to (dis‐)engage from, might often become sources of tension. Moreover, activists’ modus operandi is often to influence a general public, yet the general public may sometimes be skewed towards one or another opinion in relation to certain struggles and social issues. We take Lebanon as one such example, where identifying as LGBTQIA+ continues to be a societal taboo (LebMash, n.d.). Members of the queer community are constantly targeted by civilian groups (such as Jnoud El Rab, see Sleiman, 2023) and Lebanese authorities (Human Rights Watch, 2023), and consequently, solidarity with this group might be perceived as ‘radical’. Amidst this context, progressive activist groups struggling for a anti‐sectarian, secular country sometimes debate the extent to which they publicize their politics of solidarity with queer communities. The logic goes: In order to mobilize, politically educate, and reach more individuals in a divided, sectarian society, messages may need to be streamlined and toned down so that progressive groups are not delegitimized as ‘too radical’. Alternatively, groups may decide to nonetheless practice their pro‐LGBTQIA+ politics behind‐the‐scenes, by engaging their members in volunteering efforts to provide shelter, food and other covert support for LGBTQIA+ individuals and activists. Adopting a collective liberation mindset may problematize such exclusionary practices and increase these contradictions, leading to tensions among group members.

Similarly, we ponder of the extent to which adopting a collective liberation mindset itself requires a certain amount of privilege. In other words, while collective liberation inherently places equal value to all struggles for the liberation of all people, oppressed peoples inevitably, often unconsciously, create a hierarchy of struggles. For instance, individuals in immediate risk (e.g., wars, political turmoil, cost‐of‐living crises) may trivialize other struggles (e.g., queer identity, climate change). In this way, it would also be interesting for future research to explore the prevalence and differing understandings, of collective liberation between peoples of the Global South and those situated within the imperial core. Finally, the usual tensions of political organizing are also inevitable. For example, conflicts may arise regarding political strategies and tactics towards groups’ potentially differing objectives, despite a single point of unity (liberation). This is compounded by the inevitable differences in values/norms between individuals (Beamish & Luebbers, 2009; Daphi et al., 2022). However, these tensions, described by Mao Tse‐tung (1957) as *contradictions between ourselves* (i.e., *among the people*), are an inevitable part of political organizing that ensure internal progress and new unity within the movement. Although we highlighted potential challenges when practising collective liberation, we draw these reflections from our own lived experiences and the preliminary evidence that we could find in the literature. Consequently, future research may investigate these tensions further, exploring the conditions under which they may be opportunities for growth or act as obstructions for political organizing and forming alliances.

As a final note, we note that although we discuss collective liberation in relation to the collective action literature, many of the points raised also apply and are relevant to other areas of social psychological research, such as intergroup conflict. We encourage researchers from these subfields to use this paper as a starting point to reflect on their own research practices.

## CONCLUSION

This paper represents a small and humble attempt to bring forth the framework and concept of collective liberation into our research and organizing practices as scholars, within the word limits of a typical journal article that does not provide space for such in‐depth conversations. We hope it sparks discussions within our discipline and among one another as scholars, and that future work begins to explore the vast avenue of, and offered by, collective liberation.

## AUTHOR CONTRIBUTIONS

**Julia A. Schreiber:** Conceptualization; investigation; writing – original draft; writing – review and editing. **Yara Zebian:** Conceptualization; project administration; writing – original draft; writing – review and editing. **Mete S. Uysal:** Conceptualization; writing – review and editing; writing – original draft.

## FUNDING INFORMATION

We did not receive any financial support for the research, the authorship and/or the publication of this article.

## CONFLICT OF INTEREST STATEMENT

The author(s) declared no potential conflicts of interest with respect to the authorship, and/or publication of this article.

## Data Availability

Data derived from public domain resources.

## Footnotes

^1^
This allyship has previously been questioned by Energy (2023), who emphasized that “the links between Grenfell and Gaza… are materially intertwined”. As an illustration, we note that the Council of the Royal Borough of Kensington and Chelsea (i.e., the local authorities), whose indifference and neglect enabled the Grenfell fire crime (Booth & Sinmaz, 2024), are directly complicit in the genocide of Palestinians. They invest pension contributions into arms companies that manufacture weapons which murder Palestinians (e.g., Lockhead Martin, BAE Systems).
